# Female sexual dysfunction in premenopausal women with diabetes mellitus in northern Nigeria: A cross-sectional study from two tertiary hospitals examining associations with glycaemic control and reproductive hormones

**DOI:** 10.1371/journal.pone.0361503

**Published:** 2026-11-02

**Authors:** Fatima M. Damagum, Amina H. Hassan, Andrew E. Uloko, Hauwa A. Habeeb, Hadiza B. Damagum, Zainab A. Umar, Hassan A. Murtala, Zainab Abdulkadir, Ibrahim Y. Ibrahim, Zainab U. Ibrahim, Umar F. Abdullahi, Hamisu M. Salihu, Muktar H. Aliyu, Phylis J. Kanki, Baba M. Musa

**Affiliations:** 1 Department of Family Medicine, Aminu Kano Teaching Hospital (AKTH), Kano, Nigeria; 2 Department of Internal medicine, Aminu Kano Teaching Hospital (AKTH), Kano, Nigeria; 3 Faculty of Clinical Sciences, Bayero University, Kano, Nigeria; 4 Department of Epidemiology and Population Health, Kano Independent Research Centre Trust, Kano, Nigeria; 5 Department of Maths and statistics, Bayero University Kano, Nigeria; 6 Vanderbilt Institute for Global Health, Vanderbilt University Medical Centre (VUMC), Nashville, Tennessee, United States of America; 7 Department of Immunology and Infectious Diseases, Harvard T.H. Chan School of Public Health, Boston, United States of America; Public Library of Science, UNITED KINGDOM OF GREAT BRITAIN AND NORTHERN IRELAND

## Abstract

**Background:**

Female sexual dysfunction (FSD) is a recognised complication of diabetes mellitus (DM), but evidence on its clinical and biological correlates in sub-Saharan Africa remains limited. FSD is defined as a disorder characterized by disturbances in sexual desire, arousal, orgasm, and/or pain that causes interpersonal difficulty or distress. This study examined associations between glycaemic control, reproductive hormonal status, and FSD among premenopausal women with diabetes in northern Nigeria.

**Methods:**

We conducted a cross-sectional study among 180 premenopausal women aged 18–45 years attending diabetes clinics at two tertiary hospitals in Kano, Nigeria. Sexual function was assessed using the Female Sexual Function Index (FSFI), with scores ≤26 indicating FSD. Glycaemic control was measured using glycated haemoglobin (HbA1c), categorised as optimal (≤7.0%) or suboptimal (>7.0%). Reproductive hormones (FSH, LH, Oestrogen, testosterone, prolactin, and progesterone) were measured at phase-specific points of the menstrual cycle. Multivariable logistic regression models were used to assess associations with FSD, and linear regression models were used to examine associations with FSFI domain scores.

**Results:**

Overall, 51.7% of participants screened positive for FSD. Suboptimal glycaemic control was associated with higher odds of FSD (adjusted odds ratio [AOR] 2.80; 95% CI 1.05–7.72). It was also associated with lower orgasm (β −0.77; p = 0.032) and satisfaction (β −0.88; p = 0.003) scores. No statistically significant associations were observed between reproductive hormonal imbalance (including FSH, LH, Oestrogen, testosterone, prolactin, and progesterone) and FSD or FSFI domain scores.

**Conclusions:**

FSD was common among premenopausal women with diabetes in this setting. Suboptimal glycaemic control was associated with FSD and with lower orgasm and satisfaction scores, while reproductive hormonal measures were not associated with FSD. These findings provide evidence on clinical correlates of FSD in a sub-Saharan African population.

## Introduction

Diabetes mellitus (DM) is a prevalent and growing global public health concern. The rising number of people with DM is driven by population growth, aging, urbanization, and increasing rates of obesity and physical inactivity [1]. Approximately 11.1% of adults (20–79 years) or 1 in 9 individuals are living with diabetes, with over 40% remaining undiagnosed [2]. Projections indicate a 46% increase in global DM prevalence by 2050, affecting 1 in 8 adults (853 million people) [2,3]. The International Diabetes Federation (IDF) Diabetes Atlas 2025 projects a 46% increase in global DM prevalence by 2050, affecting 1 in 8 adults (853 million people) [2,3].

The burden of diabetes is also increasing in sub-Saharan Africa, including Nigeria. In 2019, 3.9 million adults in Nigeria had diabetes, a figure expected to rise to 6.0 million by 2045 [4,5]. A 2024 systematic review and meta-analysis revealed that the prevalence of type 2 diabetes in Nigeria (7.0%) was nearly double previous IDF estimates (3.7%) marking a 21.3% increase since 2019 [2–4].

DM is a metabolic disorder characterized by chronic hyperglycaemia, leading to systemic multiorgan damage and progressive microvascular and macrovascular complications [5]. Beyond its well-documented medical and psychological effects, DM also contributes to sexual dysfunction [5–7]. However, while male sexual dysfunction is a well-recognized complication of DM, Female Sexual Dysfunction (FSD) remains understudied and often overlooked, despite evidence showing that women with both type 1 and type 2 DM experience sexual dysfunction at least twice as compared to those without DM [8–11].

FSD is a common but stigmatized worldwide health concern, characterized by disturbances in sexual desire, arousal, orgasm, and pain, collectively termed hypoactive sexual desire disorder, sexual arousal disorder, orgasmic disorder, and genito-pelvic pain/penetration disorder [12]. These can occur singly or in combination and are further categorized as primary FSD, when no medical or psychiatric cause can be identified or as secondary FSD results when due to recognized medical conditions, medications, or psychological factors [12,13]. The pathophysiological links between DM and FSD include chronic hyperglycaemia-induced microvascular disease, peripheral neuropathy affecting genital sensation and arousal, autonomic dysfunction impairing lubrication, and disturbances in hormonal regulation affecting desire and orgasmic response [7,10,11,13].

A normal sexual response depends on an intact sensory and autonomic nervous system, which triggers smooth-muscle relaxation and increased genital blood flow [13]. In women with type 2 DM, FSD is believed to arise through several mechanisms, including chronic hyperglycaemia, microvascular disease, peripheral neuropathy, and disturbances in hormonal regulation [7,10,11,13]. However, despite these proposed pathways, the precise biological processes linking DM to FSD remain poorly understood, and the relative contribution of glycaemic dysregulation versus hormonal imbalance is yet to be clearly established.

Globally, the prevalence of FSD in the general population ranges from 26% to 60%, with higher rates reported among women with chronic conditions [14]. In sub-Saharan Africa, limited data suggest FSD prevalence of 35--55% among women attending gynaecology and family planning clinics, though population-based estimates are scarce [15–18]. Among women with DM specifically, global prevalence estimates range from 40% to 80%, yet data from African settings remain particularly sparse [18,19]. This gap is concerning given the rising burden of DM and high prevalence of obesity and gestational DM in sub-Saharan Africa.

Furthermore, gender inequality, a pervasive social phenomenon characterized by unequal access to rights, opportunities, and resources based on gender, continues to shape research priorities [20]. Sexual dysfunction research in DM disproportionately focuses on men; a cursory PubMed search for “female sexual dysfunction AND diabetes” yielded fewer than 150 publications, compared with nearly 4,000 for male sexual dysfunction.

As care for DM becomes increasingly patient-centred and holistic, attention to sexual health should be routinely provided. This study aims to clarify the relationship between FSD, glycaemic control, and hormonal profiles (FSH, LH, oestrogen, testosterone, prolactin, and progesterone) among women with DM in Nigeria. Our findings contribute evidence from an understudied population and seek to inform more comprehensive clinical practice. Continued research in this neglected domain is critical to improving the quality of life and overall well-being of the millions of women living with DM worldwide.

## Methods

### Study design/site

This was a cross-sectional study conducted at the endocrinology clinics of Aminu Kano Teaching Hospital (AKTH) and Murtala Muhammad Specialist Hospital (MMSH) in Kano, northwestern Nigeria. Both hospitals are major tertiary referral centres serving a catchment population of over 20 million people from Kano State, neighbouring states, and neighbouring countries. The endocrinology clinics in both facilities are held weekly and staffed by multidisciplinary teams comprising consultants, senior registrars, and registrars.

### Study population and sampling

Women attending the DM clinics at AKTH and MMSH during the study period were assessed for eligibility. A convenience sampling approach was employed, whereby all consecutive eligible women presenting to the clinics during the data collection period were invited to participate until the required sample size was achieved. This approach reduced investigator-driven selection and was feasible within routine clinical care. While probability sampling would have been preferable for representativeness, clinic attendance patterns and the sensitive nature of sexual health research in this cultural context made consecutive sampling the most practical approach to achieve adequate recruitment while minimizing selection bias. Data collection and participant recruitment were conducted from 2^nd^ December 2024–27^th^ June 2025.

### Eligibility criteria

Participants were eligible for inclusion if they met all the following criteria: a) Women diagnosed with DM for at least six months b) Aged 18–45 years. c) Women with regular menstrual cycles and d) Sexually active women in a heterosexual marriage or partnership.

Women were excluded if they: a) Were pregnant at the time of recruitment) Had reached menopause, defined as the absence of menstrual bleeding for 12 consecutive months. c) Had undergone bilateral oophorectomy or hysterectomy. d) Were currently breastfeeding.

### Ethics

All participants provided written informed consent prior to enrolment. Consent was obtained by trained research assistants and documented using signed consent forms. Ethical approval for this study was obtained from the AKTH Research Ethics Committee (approval number: NHREC/28/01/2020/AKTH/EC/3887) and the Kano Ministry of Health Institutional Review Board (approval number: NHREC/17/03//2018). Participants who screened positive for FSD or had hormonal imbalances were counselled by the research team and referred to the family medicine and endocrinology clinics for further evaluation and management, including sexual counselling and endocrine optimisation, as part of standard care at no cost. No participant was denied care based on study participation.

### Sample size determination

The required sample size was estimated to be between 170 and 190 participants, based on a reported FSD prevalence rate of 86.0% among women with DM attending AKTH [21]. This prevalence estimate was derived from a previous cross-sectional survey conducted at the same institution using the same FSFI instrument and cutoff score. The sample size provided adequate precision for prevalence estimation and sufficient power for exploratory association analyses.

### Data collection

Data were collected using interviewer-administered questionnaires deployed via the RED Cap mobile application [22]. Trained research assistants obtained information on socio-demographic characteristics, reproductive history, clinical variables, and DM-related factors.

**Study procedure:** Participants attended two or three visits depending on their cycle. At visit 1 (recruitment day), written informed consent was obtained, the questionnaire was administered, and venous blood was collected for HbA1c measurement. A menstrual history was taken to schedule visit 2. At visit 2 (phase-specific day: follicular day 3 or luteal day 21), venous blood (5 mL) was collected for hormone assays. A third visit was scheduled as needed to obtain blood samples on exact dates of menstrual cycle. Follow-up telephone calls were made to remind participants of appointment dates.

The Female Sexual Function Index (FSFI) is a validated 19-item questionnaire that evaluates six domains: desire, arousal, lubrication, orgasm, satisfaction, and pain. FSFI total scores range from 2 to 36, with scores ≤26 indicating a positive screen for FSD [23]. The FSFI does not establish a clinical diagnosis but serves as a validated screening instrument for sexual function impairment. The Hausa-language version of the FSFI, developed through forward translation into Hausa by bilingual linguists, back translation into English by independent translators, and pilot testing among twenty Hausa speaking women with DM for cultural appropriateness and comprehension, was used for participants with limited English proficiency. The translation process followed WHO guidelines for instrument adaptation and was approved by the FSFI copyright holders.

A detailed menstrual history was obtained to estimate individual cycle length and determine follicular and luteal phases. Participants were scheduled for venous blood sampling during specific phases of their menstrual cycle. Follicle-stimulating hormone (FSH), luteinizing hormone (LH), oestrogen, testosterone, and prolactin during the follicular phase (Day 3) and progesterone during the luteal phase (Day 21 or seven days prior to the expected next menstrual period)

Blood sample (5mls) for hormone measurement were collected. Serum was aliquoted and stored at −20 °C until analysis. Hormone concentrations were measured using a Cobas e411 analyser (Roche Diagnostics). Reference ranges for premenopausal women were: FSH 3.5–12.5 IU/L (follicular), LH 2.4–12.6 IU/L, oestradiol 90–716 pmol/L, testosterone 0.4–2.1 nmol/L, prolactin 109–557 mIU/L, and luteal-phase progesterone 5.3–86.0 nmol/L [24]. Overall hormonal status was categorised as normal or hormonal imbalance, defined as the presence of two or more reproductive hormones outside the respective reference range.

Glycaemic control was assessed using glycated haemoglobin (HbA1c). A 3 mL venous blood sample was collected. For participants who had HbA1c measured within four weeks prior to enrolment as part of routine clinical care, those results were used; all others had fresh HbA1c measurements performed for the study. HbA1c was measured using high-performance liquid chromatography (HPLC) on a Bio-Rad D-100 analyser (Bio-Rad Laboratories, Hercules, CA, USA).

Diabetes-related complications (nephropathy, neuropathy, retinopathy) were assessed through review of medical records, participant self-report of diagnosed complications, and clinical examination findings documented in clinic notes. Microvascular complications were defined according to standard clinical criteria: nephropathy as albuminuria (>30 mg/g creatinine) or estimated glomerular filtration rate <60 mL/min/1.73m2; neuropathy as presence of symptoms (numbness, tingling, pain) plus clinical examination findings (reduced vibration perception or monofilament testing); retinopathy as documented on ophthalmologic examination.^24^

Participants who required follow-up for test results (hormonal profiles or HbA1c) were contacted by telephone within 2 weeks of sample collection. Those with abnormal results were invited to return to the clinic for review and management. All participants received their results and appropriate counselling regardless of study completion.

### Statistical Analysis

Data analysis was performed using R statistical software (v 4.5.2; R Core Team 2025). Glycaemic control was categorized as optimal (HbA1c ≤ 7.0%) or suboptimal (HbA1c > 7.0%). FSD was analysed as a binary outcome based on FSFI screening status; screened positive for FSD vs screened negative for FSD.

Continuous variables were summarized using medians and interquartile ranges, while categorical variables were summarized as frequencies and percentages. Bivariate analyses employed Wilcoxon rank-sum tests for continuous variables and Pearson’s chi-squared or Fisher’s exact tests for categorical variables, as appropriate. FSFI domain score distributions by glycaemic control status were visualized using box plots.

Directed acyclic graphs (DAGs) were constructed a priori to guide confounder selection for multivariable logistic regression models examining (a) the association between glycaemic control and screening positive for female sexual dysfunction (FSD) and (b) the association between overall hormonal status and screening positive for FSD (Fig 1).

**Fig 1:**
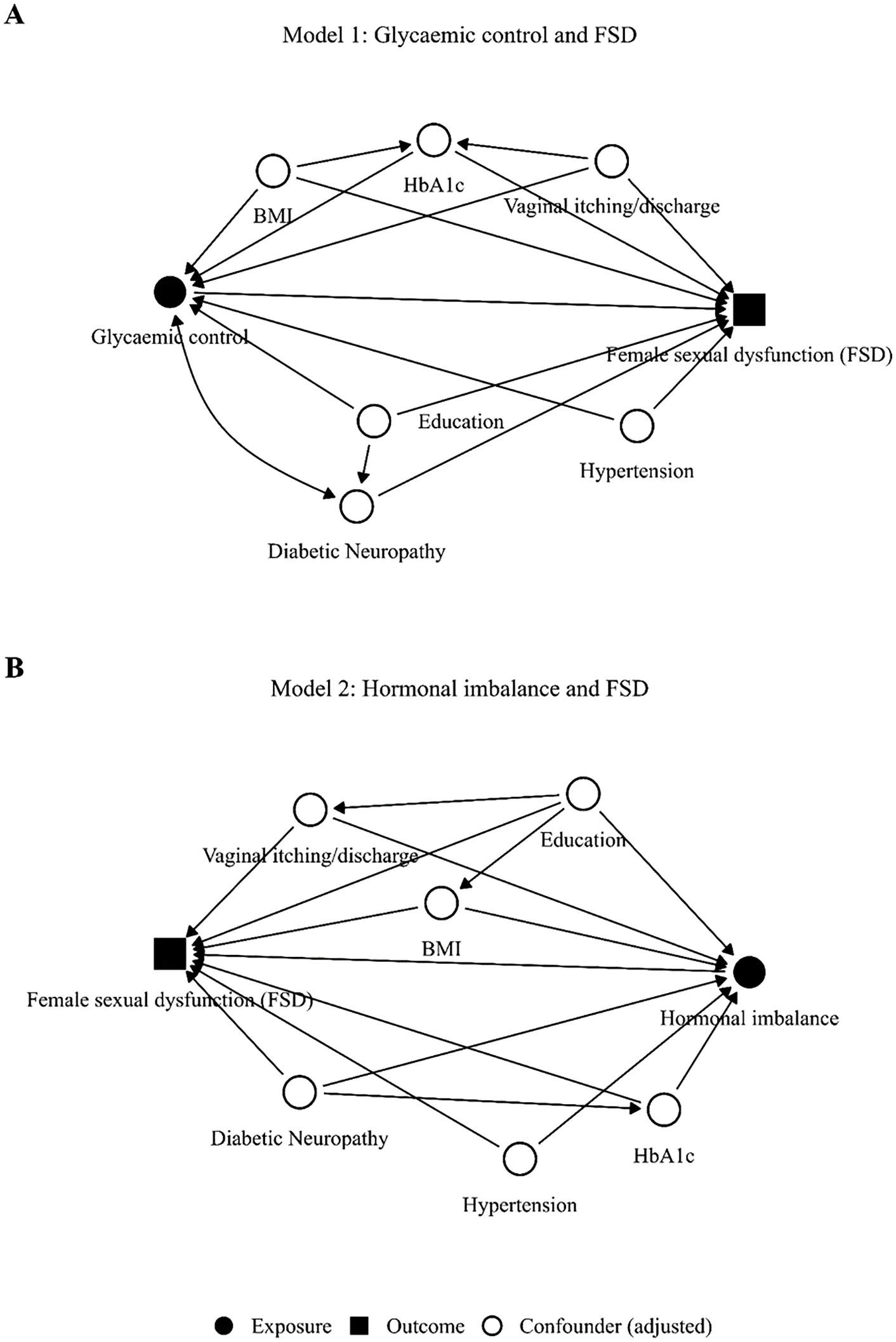
Directed acyclic graphs (DAGs) illustrating hypothesized causal pathways for the development of FSD among women with DM, Kano, Nigeria.

Adjusted models accounted for socio-demographic, clinical, and reproductive variables identified through the DAGs. Additional subgroup analyses using linear regression models assessed associations between glycaemic control and individual FSFI domain scores. Statistical significance was set at *p* < 0.05.

In the multivariable logistic regression model examining glycaemic control and FSD (Model 1), we included both categorical glycaemic control (optimal vs. suboptimal) and continuous HbA1c to assess both the clinically relevant threshold effect and the dose-response relationship. The categorical variable captures the clinically actionable cutoff of 7%, while the continuous variable adjusts for linear trends within categories. We verified that multicollinearity was not problematic (variance inflation factor <2.0 for both variables). As a sensitivity analysis, we also fitted models with only categorical or only continuous HbA1c; the direction and significance of associations remained consistent.

## Results

A total of 180 eligible women were recruited. Of these, 93 participants (51.7%) screened positive for FSD based on an FSFI total score ≤26, while 87 participants (48.3%) screened negative for FSD (Fig 2).

**Fig 2:**
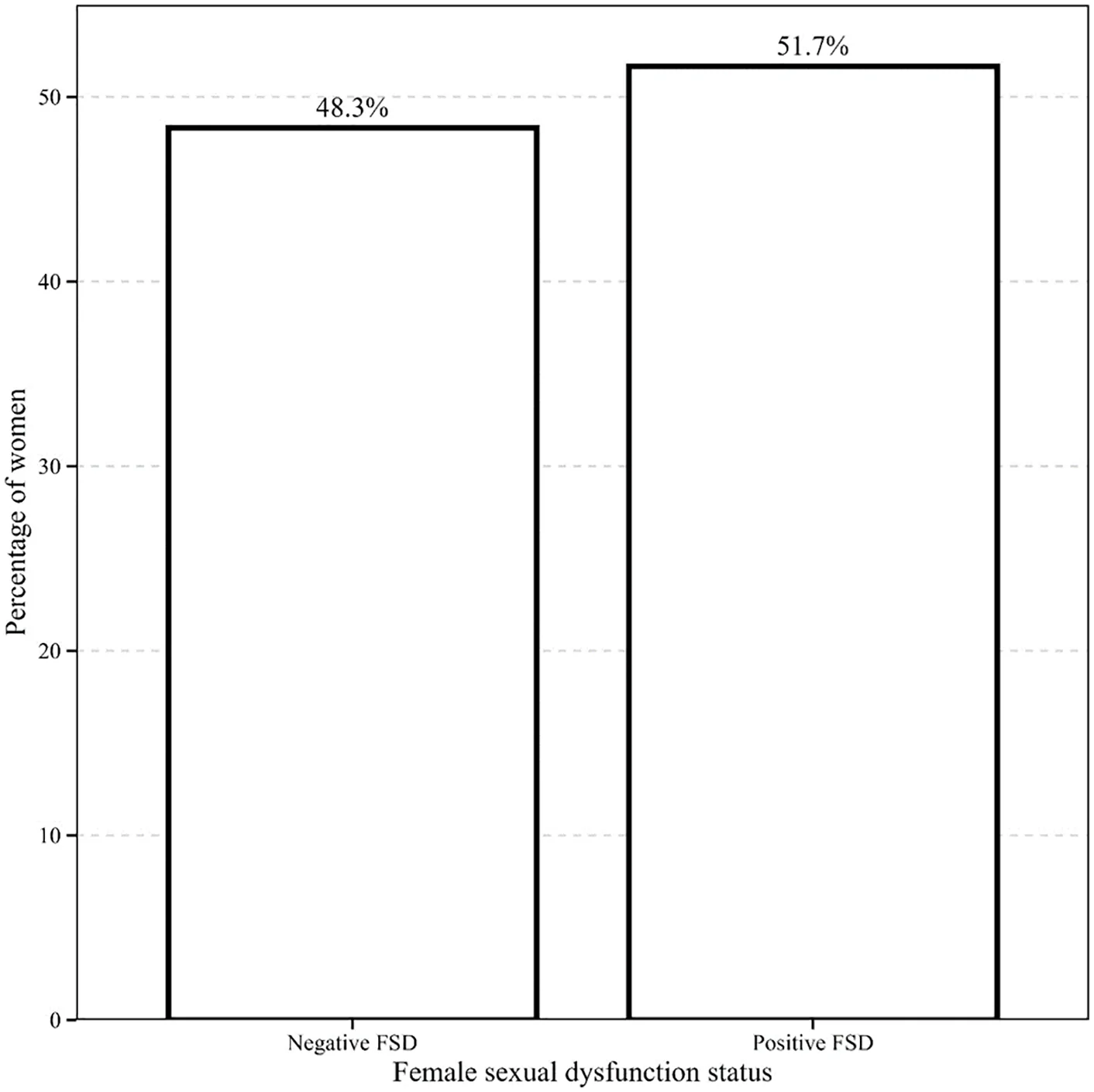
Prevalence of Female Sexual Dysfunction Among Women with DM.

The majority of participants were aged 35–45 years, with a comparable median age of 40.0 years among women who screened negative for FSD (IQR: 37.0–43.0) and those who screened positive (IQR: 36.0–43.0) (Table 1). All participants were married and predominantly of Hausa ethnicity; 83% among FSD-negative and 81% among FSD-positive women.

**Table 1:** Baseline sociodemographic and clinical characteristics among women with DM stratified by FSD status, Kano, Nigeria.

Characteristic	Negative FSD (N = 87)	Positive FSD (N = 93)	p-value
Age (Years), Median (Q1-Q3)	40.0 (37.0-43.0)	40.0 (36.0-43.0)	0.9^*1*^
(Missing)	4	1	
Age group, n (%)			0.077^2^
18-24	0 (0%)	5 (5.4%)	
25-34	12 (14%)	9 (9.8%)	
35-45	71 (86%)	78 (85%)	
Ethnicity, n (%)			0.4^2^
Hausa	72 (83%)	75 (81%)	
Fulani	13 (15%)	12 (13%)	
Others	2 (2.3%)	6 (6.5%)	
Marital status, n (%)			>0.9^2^
Single	0 (0%)	0 (0%)	
Married	87 (100%)	93 (100%)	
Education, n (%)			0.056^3^
Primary	14 (16%)	25 (27%)	
Secondary	42 (48%)	30 (33%)	
Tertiary	25 (29%)	24 (26%)	
Others	6 (6.9%)	13 (14%)	
(Missing)	0	1	
Monthly income, n (%)			0.14^3^
< 18,000	23 (26%)	34 (37%)	
18,000-50,000	26 (30%)	27 (29%)	
>50,000-150,000	17 (20%)	21 (23%)	
>150,000	21 (24%)	11 (12%)	
Occupation, n (%)			0.7^3^
Employed	44 (51%)	50 (54%)	
Unemployed	43 (49%)	43 (46%)	
Gravidity, n (%)			0.4^3^
1–3	14 (16%)	17 (18%)	
4–5	20 (23%)	14 (15%)	
>5	53 (61%)	62 (67%)	
Parity, n (%)			0.082^3^
<5	41 (47%)	32 (34%)	
≥5	46 (53%)	61 (66%)	

^*1*^Wilcoxon rank sum test; ^2^Fisher’s exact test; ^3^Pearson’s Chi-squared test.

Educational attainment did not differ significantly by FSD screening status. Similarly, no statistically significant differences were observed between the two groups with respect to monthly income, occupation, gravidity, parity, history of vaginal discharge, genital cutting, duration of DM, use of oral hypoglycaemic agents or insulin, DM-related complications, anthropometric measurements, or blood pressure parameters (Table 2). Similarly, reproductive hormone profiles did not differ significantly between women who screened positive for FSD and those who screened negative. Specifically, no statistically significant associations were observed between FSD screening status and levels of prolactin, FSH, LH, oestrogen, testosterone, or luteal-phase progesterone, nor with overall hormonal imbalance (Table 3). Although the median HbA1c was higher in the FSD-negative group (9.1%) than in the FSD-positive group (8.3%), this difference was not statistically significant (p = 0.5). The association between suboptimal glycaemic control and FSD emerged only after adjusting for confounders in multivariable analysis, suggesting that other factors (e.g., education, BMI, comorbidities) modified the crude relationship.

**Table 2:** Clinical and diabetes-related characteristics among women with diabetes mellitus stratified by FSD status, Kano, Nigeria.

Characteristics	Negative FSD (N = 87)	Positive FSD (N = 93)	p-value
Vaginal itching/discharge			0.4^3^
No	66 (81%)	65 (76%)	
Yes	15 (19%)	20 (24%)	
(Missing)	6	8	
Genital cutting, n (%)			0.5^2^
No	81 (96%)	85 (93%)	
Yes	3 (3.6%)	6 (6.6%)	
(Missing)	3	2	
Duration of DM, n (%)			0.2^3^
<10 years	54 (63%)	66 (71%)	
≥10 years	32 (37%)	27 (29%)	
(Missing)	1	0	
Oral DM medication, n (%)			0.7^3^
No	6 (7.0%)	5 (5.4%)	
Yes	80 (93%)	87 (95%)	
(Missing)	1	1	
Insulin use, n (%)			0.4^3^
No	59 (73%)	65 (78%)	
Yes	22 (27%)	18 (22%)	
(Missing)	6	10	
Waist circumference, Median (Q1-Q3)	94 (87-100)	93 (86-102)	0.4^*1*^
(Missing)	14	18	
BMI, n (%)			0.4^2^
<18.5 Underweight	3 (3.5%)	1 (1.1%)	
18.5-24.9 Normal	21 (25%)	22 (24%)	
25.0–29.9 Overweight	38 (45%)	35 (38%)	
≥30 Obese	23 (27%)	34 (37%)	
(Missing)	2	1	
DM complication: None, n (%)			0.082^3^
No	45 (52%)	60 (65%)	
Yes	42 (48%)	33 (35%)	
DM complication: Nephropathy, n (%)			0.3^2^
No	85 (98%)	87 (94%)	
Yes	2 (2.3%)	6 (6.5%)	
DM complication: Neuropathy, n (%)			0.12^3^
No	55 (63%)	48 (52%)	
Yes	32 (37%)	45 (48%)	
DM complication: Other, n (%)			0.7^3^
No	71 (82%)	74 (80%)	
Yes	16 (18%)	19 (20%)	
Comorbidity: None, n (%)			0.10^3^
No	38 (44%)	52 (56%)	
Yes	49 (56%)	41 (44%)	
Comorbidity: Hypertension, n (%)			0.4^3^
No	52 (60%)	50 (54%)	
Yes	35 (40%)	43 (46%)	
Comorbidity: Asthma, n (%)			0.4^2^
No	85 (98%)	88 (95%)	
Yes	2 (2.3%)	5 (5.4%)	
Comorbidity: Sickle cell disease, n (%)			>0.9^2^
No	87 (100%)	93 (100%)	
Yes	0 (0%)	0 (0%)	
Comorbidity: Psychiatric illness, n (%)			0.2^2^
No	87 (100%)	90 (97%)	
Yes	0 (0%)	3 (3.2%)	
Comorbidity: Thyroid disease, n (%)			0.5^2^
No	86 (99%)	93 (100%)	
Yes	1 (1.1%)	0 (0%)	
Comorbidity: Other, n (%)			0.6^2^
No	86 (99%)	90 (97%)	
Yes	1 (1.1%)	3 (3.2%)	
Smoking, n (%)			>0.9^2^
No	87 (100%)	93 (100%)	
Yes	0 (0%)	0 (0%)	
Alcohol use, n (%)			>0.9^2^
No	86 (100%)	92 (99%)	
Yes	0 (0%)	1 (1.1%)	
(Missing)	1	0	

^*1*^ Wilcoxon rank sum test; ^2^Fisher’s exact test; ^3^Pearson’s Chi-squared test

*Missing values represent participants who declined to answer or had incomplete clinical records. Proportions are calculated based on non-missing responses

**Table 3:** Comparison of hormonal profiles and female sexual dysfunction status.

Characteristic	Negative FSD (N = 87)	Positive FSD (N = 93)	p-value
**Overall Hormonal Status, n (%)**			0.14^*1*^
Normal Hormonal Status	13 (15%)	22 (24%)	
Hormonal imbalance	74 (85%)	71 (76%)	
**Prolactin, n (%)**			0.6^*1*^
Normal	58 (67%)	65 (70%)	
prolactin Imbalance	29 (33%)	28 (30%)	
**FSH, n (%)**			0.6^*1*^
Normal	57 (66%)	57 (61%)	
FSH Imbalance	30 (34%)	36 (39%)	
**LH, n (%)**			0.5^*1*^
Normal	75 (86%)	77 (83%)	
LH Imbalance	12 (14%)	16 (17%)	
**Oestrogen, n (%)**			0.8^*1*^
Normal	41 (48%)	46 (49%)	
Oestrogen Imbalance	45 (52%)	47 (51%)	
(Missing)	1	0	
**Testosterone, n (%)**			0.083^*1*^
Normal	72 (83%)	85 (91%)	
Testosterone Imbalance	15 (17%)	8 (8.6%)	
**Luteal Phase Progesterone, n (%)**			0.3^*1*^
Normal	60 (70%)	57 (62%)	
Progesterone Imbalance	26 (30%)	35 (38%)	
(Missing)	1	1	
**HBA1C, Median (Q1-Q3)**	9.1 (6.8-10.8)	8.3 (7.1-10.8)	0.5^2^
**HbA1c > 7%**			0.4^2^
No (<=7%)	24 (28%)	32 (34%)	
Yes (>7%)	63 (72%)	61 (66%)	

^*1*^Pearson’s Chi-squared test; ^2^Wilcoxon rank sum test.

Analysis of FSFI domain scores by glycaemic control status showed that women with suboptimal glycaemic control had significantly lower scores in the orgasm (*p* = 0.047) and satisfaction (*p* = 0.035) domains compared with women with optimal glycaemic control (Fig 3). No, statistically significant differences were observed in the desire, arousal, lubrication, or pain domains.

**Fig 3:**
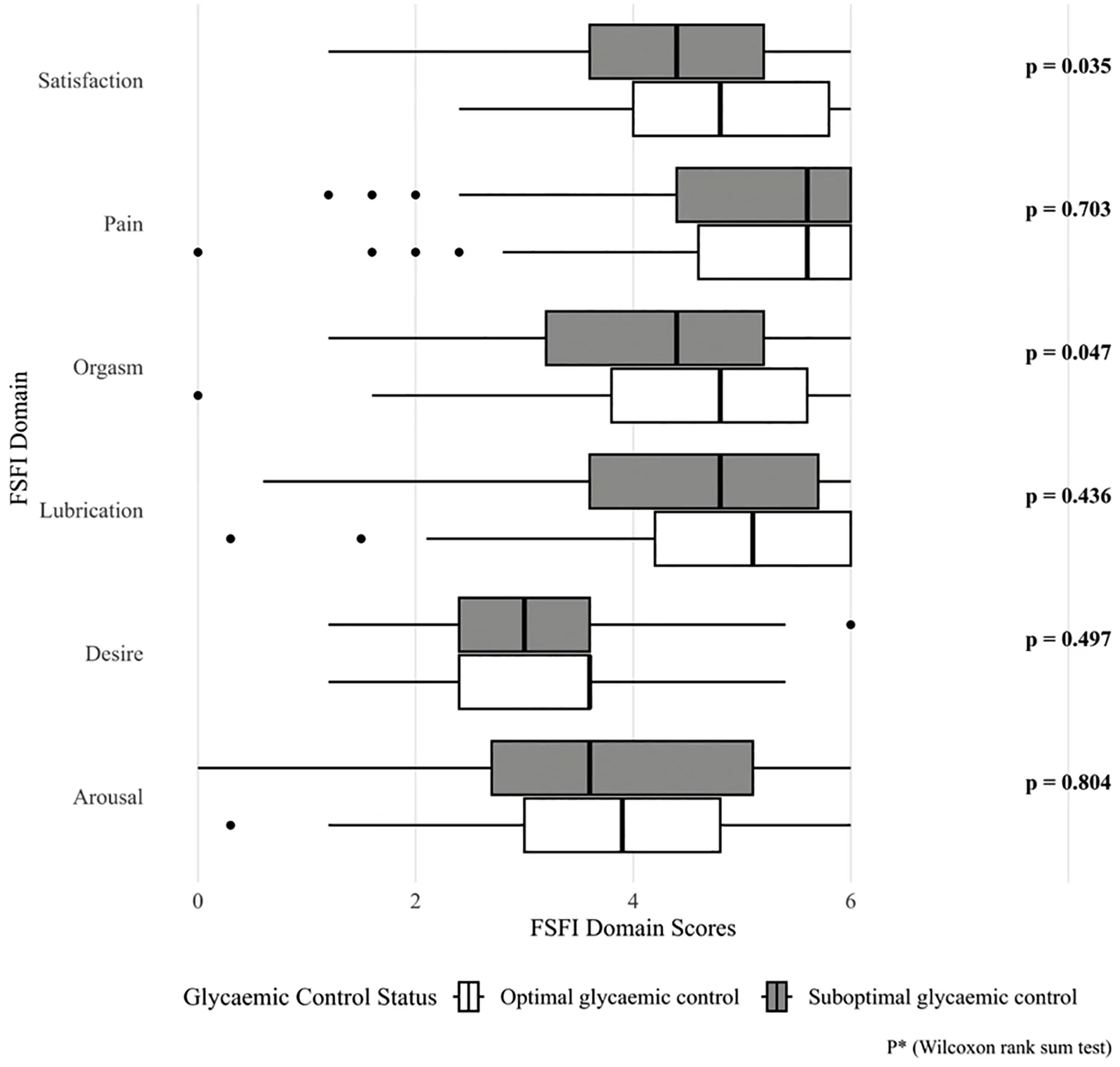
Distribution of Female Sexual Function Domain Scores by Glycaemic Control Status.

Two separate multivariable logistic regression models were fitted based on a priori directed acyclic graphs: Model 1 evaluated the association between glycaemic control and screening positive for FSD, while Model 2 evaluated the association between overall hormonal imbalance and screening positive for FSD (Table 4).

**Table 4:** Multivariable logistic regression models examining the association of glycaemic control and hormonal imbalance with female sexual dysfunction (FSD), Kano, Nigeria.

Characteristic	AOR	95% CI	p-value
Model 1: Causal relationship between glycaemic control and FSD			
Glycaemic control status			
Optimal glycaemic control	—	—	
Suboptimal glycaemic control	2.80	1.05, 7.72	0.042
HbA1c Levels, (confounder)	0.88	0.76, 1.02	0.089
Educational status, (confounder)			
Tertiary	—	—	
Primary	1.82	0.72, 4.72	0.2
Secondary	0.65	0.29, 1.47	0.3
Others	3.98	1.09, 17.2	0.046
BMI, (confounder)			
<18.5 Underweight	—	—	
18.5-24.9 Normal	4.48	0.41, 111	0.3
25.0–29.9 Overweight	4.13	0.39, 101	0.3
≥30 Obese	6.66	0.59, 169	0.2
History of vaginal itching or discharge, (confounder)			
No	—	—	
Yes	1.21	0.52, 2.83	0.7
Comorbidity: Hypertension, (confounder)			
No	—	—	
Yes	0.99	0.50, 1.94	>0.9
DM complication: Nephropathy, (confounder)			
No	—	—	
Yes	1.61	0.25, 13.3	0.6
			
Model 2: Causal relationship between hormonal imbalance and FSD			
Overall hormonal status			
Normal Hormonal Status	—	—	
Hormonal imbalance	0.46	0.19, 1.05	0.070
Educational status, (confounder)			
Tertiary	—	—	
Primary	1.57	0.62, 4.05	0.3
Secondary	0.59	0.26, 1.33	0.2
Others	3.19	0.90, 13.4	0.085
BMI, (confounder)			
<18.5 Underweight	—	—	
18.5-24.9 Normal	5.00	0.47, 123	0.2
25.0–29.9 Overweight	4.16	0.40, 102	0.3
≥30 Obese	6.67	0.61, 169	0.2
History of vaginal itching or discharge, (confounder)			
No	—	—	
Yes	1.19	0.52, 2.76	0.7
Comorbidity: Hypertension, (confounder)			
No	—	—	
Yes	0.98	0.50, 1.92	>0.9
DM complication: Nephropathy, (confounder)			
No	—	—	
Yes	1.79	0.28, 14.6	0.5
			

Abbreviations: AOR = Adjusted odds Ratio, CI = Confidence Interval, FSD = Female Sexual Dysfunction; DM = Diabetes Mellitus; BMI = Body Mass Index; HbA1c = Glycated Haemoglobin

In multivariable logistic regression models guided by directed acyclic graphs (DAGs), women with suboptimal glycaemic control were nearly three times more likely to screen positive for FSD compared with women with optimal glycaemic control (adjusted odds ratio [AOR]: 2.80; 95% confidence interval [CI]: 1.05–7.72; *p* = 0.042) after adjusting for relevant socio-demographic and clinical confounders (Table 4, Model 1).

Overall hormonal imbalance was not independently associated with screening positive for FSD after adjustment (AOR: 0.46; 95% CI: 0.19–1.05; *p* = 0.070) (Table 4, Model 2). In domain-specific multivariable linear regression analyses, suboptimal glycaemic control was associated with a 0.77-point reduction in FSFI orgasm scores (β = −0.77; 95% CI: −1.50 to −0.07; *p* = 0.032) and a 0.88-point reduction in FSFI satisfaction scores (β = −0.88; 95% CI: −1.50 to −0.30; *p* = 0.003) compared with optimal glycaemic control (Table 5). No significant associations were observed between glycaemic control and FSFI desire, arousal, lubrication, or pain domains.

**Table 5:** Causal models examining female Sexual Function Index (FSFI) domains and glycaemic control, Kano, Nigeria.

Characteristic	Beta	95% CI	p-value
Model 2b: Causal relationship between FSFI-orgasm and FSD			
Glycaemic control status			
Optimal glycaemic control	—	—	
Suboptimal glycaemic control	−0.77	−1.5, −0.07	0.032
HbA1c Levels, (confounder)	0.07	−0.03, 0.17	0.2
Educational level, (confounder)			
Tertiary	—	—	
Primary	−0.76	−1.4, −0.09	0.027
Secondary	−0.11	−0.72, 0.50	0.7
Others	−0.10	−0.97, 0.78	0.8
Duration of DM (years), (confounder)			
<10 years	—	—	
≥10 years	−0.18	−0.68, 0.32	0.5
Waist circumference (cm), (confounder)	−0.01	−0.03, 0.01	0.4
History of vaginal itching or discharge, (confounder)			
No	—	—	
Yes	0.28	−0.35, 0.91	0.4
Model 2c: Causal relationship between FSFI-satisfaction and FSD			
Glycaemic control status			
Optimal glycaemic control	—	—	
Suboptimal glycaemic control	−0.88	−1.5, −0.30	0.003
HbA1c Levels, (confounder)	0.10	0.01, 0.18	0.023
Educational level, (confounder)			
Tertiary	—	—	
Primary	−0.50	−1.1, 0.05	0.077
Secondary	0.31	−0.20, 0.81	0.2
Others	−0.06	−0.79, 0.66	0.9
Duration of DM (years), (confounder)			
<10 years	—	—	
≥10 years	0.01	−0.41, 0.42	>0.9
Waist circumference (cm), (confounder)	−0.01	−0.02, 0.01	0.4
History of vaginal itching or discharge, (confounder)			
No	—	—	
Yes	0.07	−0.45, 0.59	0.8
Model 2d: Causal relationship between FSFI-desire and FSD			
Glycaemic control status			
Optimal glycaemic control	—	—	
Suboptimal glycaemic control	0.06	−0.41, 0.53	0.8
HbA1c Levels, (confounder)	−0.03	−0.10, 0.04	0.4
Model 2e: Causal relationship between FSFI-arousal and FSD			
Glycaemic control status			
Optimal glycaemic control	—	—	
Suboptimal glycaemic control	−0.34	−0.89, 0.22	0.2
HbA1c Levels, (confounder)	0.07	−0.01, 0.15	0.11
Model 2f: Causal relationship between FSFI-lubrication and FSD			
Glycaemic control status			
Optimal glycaemic control	—	—	
Suboptimal glycaemic control	−0.36	−0.92, 0.20	0.2
HbA1c Levels, (confounder)	0.06	−0.02, 0.15	0.14
Model 2g: Causal relationship between FSFI-pain and FSD			
Glycaemic control status			
Optimal glycaemic control	—	—	
Suboptimal glycaemic control	−0.22	−0.76, 0.32	0.4
HbA1c Levels, (confounder)	0.08	0.00, 0.16	0.059

Abbreviation: AOR = Adjusted odds ratio CI = Confidence Interval, FSD = Female Sexual Dysfunction; DM = Diabetes Mellitus; BMI = Body Mass Index; HbA1c = Glycated Haemoglobin.

## Discussion

To examine the relationship between FSD, glycaemic control, and reproductive hormonal profiles among women with DM, we conducted a cross-sectional study of 180 premenopausal women attending two tertiary clinics in northern Nigeria. Overall, 51.7% of participants screened positive for FSD on the FSFI. Suboptimal glycaemic control (HbA1c > 7.0%) was associated with nearly three-fold higher odds of screening positive for FSD (adjusted odds ratio [AOR] 2.80, 95% CI 1.05–7.72) after adjustment for confounders. Suboptimal glycaemic control was specifically associated with lower orgasm and satisfaction scores, whereas overall reproductive hormone abnormalities were not independently associated with screening positive for FSD.

Sociodemographic and clinical characteristics were broadly similar between women with and without FSD. Age distribution, ethnicity, marital status, income, occupation, gravidity, parity, body mass index, DM duration, treatment modality and documented microvascular complications did not differ significantly by FSD status. This pattern mirrors findings from observational studies in women with type 1 and type 2 DM, where traditional biomedical risk factors explain only part of the variance in sexual function, and psychosocial variables including depression, relationship quality and cultural norms, play a major role [8–10].

The prevalence of FSFI-screened sexual dysfunction in our sample (51.7%) is consistent with evidence indicating that women with DM experience high rates of sexual dysfunction and aligns with global estimates of elevated sexual dysfunction rates among people with DM generally [6,19]. However, direct comparisons across studies should be interpreted cautiously, as differences in measurement instruments, scoring thresholds, and population characteristics may influence prevalence estimates. These findings add to the sparse literature on female sexual dysfunction in sub-Saharan Africa.

A key contribution of this study is the demonstration of an independent association between suboptimal glycaemic control and FSD. Women with HbA1c > 7.0% were almost three times more likely to screen positive for FSD compared with those with optimal glycaemic control, even after adjustment for multiple socio-demographic and clinical confounders. This association likely reflects the cumulative effects of chronic hyperglycaemia on microvascular and neurological function rather than acute glucose fluctuations. The finding is biologically plausible: sustained hyperglycaemia impairs endothelial-dependent vasodilation and reduces genital blood flow, while diabetic neuropathy diminishes sensory feedback necessary for arousal and orgasm [19,25,26].

Domain-specific analyses further showed that suboptimal control was associated with significantly lower orgasm and satisfaction scores, while desire, arousal, lubrication and pain were not significantly different by glycaemic status. These results are biologically plausible given established links between chronic hyperglycaemia, microvascular disease and peripheral neuropathy, and impairment of the neurovascular pathways that mediate genital engorgement and orgasmic response in women [25,27]. The specificity of effects on orgasm and satisfaction (rather than desire or arousal) suggests that glycaemic control may operate primarily through peripheral vascular and neurological mechanisms rather than central hormonal or motivational pathways.

In contrast to the strong association with glycaemic control, overall reproductive hormonal imbalance was not independently related to FSD in the adjusted model (AOR 0.46, 95% CI 0.19–1.05). Individual hormones, including oestrogen, progesterone, testosterone, FSH, LH and prolactin, also did not differ significantly by FSD status. This finding appears to deviate from mechanistic models that emphasize disturbances in sex steroid and gonadotropin levels as potential mediators of FSD in DM [27,28].

Prior clinical literature has been inconsistent: some studies report associations between androgen or oestrogen abnormalities and specific FSD domains, whereas others found psychosocial factors, vascular complications and mood disorders are more salient determinants of sexual function in women with DM [29–32]. For example, a systematic review by Pontiroli et al. found that FSD in women with type 1 and type 2 DM was more strongly associated with depression and marital dissatisfaction than with metabolic control, while a more recent meta-analysis by Hashim et al. reported that poor glycaemic control was consistently associated with FSD across 12 observational studies, though effect sizes varied considerably (OR 1.4–3.2) [8,19]. The lack of a clear hormonal effect in this study may be due to the young, premenopausal age group, the timing of sample collection in the menstrual cycle, or stronger influences from non-hormonal factors like neuropathy, vascular disease, and relationship issues.

The apparent inconsistency between the crude HbA1c distribution (higher median in FSD-negative women: 9.1% vs 8.3%) and the adjusted association between suboptimal control and FSD (AOR 2.80) warrants explanation. In unadjusted comparisons, women with FSD were slightly younger and more likely to have higher education--factors associated with better healthcare engagement and thus more intensive glycaemic monitoring, which may artifactually lower HbA1c in the FSD-positive group. After adjustment for age, education, BMI, and complications, the true deleterious effect of sustained hyperglycaemia on sexual function becomes apparent. This pattern highlights the importance of multivariable adjustment in observational studies and highlights that crude comparisons may be confounded by differential healthcare access and health-seeking behaviour.

This study has several strengths. The use of a validated, multidomain instrument (FSFI) to assess sexual function, including a culturally adapted Hausa-language version, enhances internal validity and inclusivity of women with limited English proficiency. In addition, objective measurement of HbA1c and phase-specific reproductive hormones, as well as the use of directed acyclic graphs to guide confounder selection for regression models, also strengthen causal interpretation of the associations observed.

A limitation of our study is the cross-sectional design that precludes establishing temporal precedence between poor glycaemic control and FSD, raising the possibility of bidirectional relationships in which sexual distress and relationship strain may also impair self-management and metabolic control. Additionally, the absence of a non-diabetic control group precludes direct comparison of FSD prevalence between women with and without DM, which would have strengthened causal inference. Furthermore, although we timed hormonal sampling to menstrual phases, single time-point hormone measurements are subject to intra-individual variability and may misclassify endocrine status. In addition, clinic-based sampling can over-represent women with more severe or complicated DM, potentially inflating prevalence estimates. Finally, potential confounders such as depressive symptoms, partner sexual dysfunction and detailed relationship measures were not systematically assessed.

### Implications for clinical care and policy

Our findings have important clinical and public health implications. They support a model in which FSD among women with DM in this setting is driven more by chronic glycaemic dysregulation and its complications than by overt reproductive hormonal derangements, at least in the premenopausal age range studied. Our report is consistent with contemporary reviews advocating that sexual dysfunction in women with DM be approached as an integral part of the chronic complication spectrum, requiring the same level of attention as cardiovascular, renal or retinal disease. Routine enquiry about sexual health, use of brief validated tools such as the FSFI, and multidisciplinary management that addresses glycaemic optimisation, neuropathic and vascular complications, psychological distress and relational factors should be incorporated into DM care, particularly for women, whose sexual health has historically been neglected in both research and clinical practice.

### Directions for future research

Future longitudinal studies in diverse African settings are needed to clarify temporal relationships between glycaemic trajectories, evolving DM complications and specific FSD domains, and to disentangle the relative contributions of hormonal, vascular, neuropathic and psychosocial pathways. Intervention trials are needed that would test whether sustained improvement in glycaemic control, combined with targeted sexual counselling or couple-based therapy, can meaningfully improve orgasm and satisfaction scores. Such studies would provide critical evidence to guide comprehensive, gender-sensitive DM care

## Conclusion

In this cross-sectional study of premenopausal women with DM attending two tertiary hospitals in Northern Nigeria over half screened positive for FSD on the FSFI. Suboptimal glycaemic control (HbA1c > 7%) was associated with higher odds of screening positive for FSD (AOR 2.80, 95% CI 1.05–7.72) as well as lower orgasm and satisfaction domain scores. Reproductive hormonal imbalance (FSH, LH, oestrogen, testosterone, prolactin, progesterone) measured at single time points did not independently predict FSFI-screened dysfunction. These findings emphasize the multifactorial nature of sexual dysfunction in women with DM and support the integration of routine sexual health assessment into DM care. In northern Nigeria where the burden of DM is rapidly rising and sexual health concerns remain highly stigmatized, addressing FSD within DM services represents an important, contextually relevant public health opportunity to improve women’s quality of life and holistic DM outcomes.
