# Supplementary material for: Female sexual dysfunction in premenopausal women with diabetes mellitus in northern Nigeria: A cross-sectional study from two tertiary hospitals examining associations with glycaemic control and reproductive hormones
Source: PLoS One. 2026 Nov 2;21(11):e0361503. doi: 10.1371/journal.pone.0361503 (PMC13726980; doi:10.1371/journal.pone.0361503)
Supplement: Supplementary file 1 [file pone.0361503.s002.docx]

**Dear Academic Editor,**

Thank you for the opportunity to revise our manuscript. We are grateful for the thoughtful and constructive feedback provided by the reviewers. We have addressed all comments point-by-point in the table below and revised the manuscript accordingly. All changes are highlighted in the revised manuscript using track changes.

A. **Response to Journal Requirements**

| Requirement | Action Taken |
| --- | --- |
| 1.PLOS ONE style requirements | The manuscript has been formatted according to the PLOS ONE templates, including file naming, headings, and reference style. |
| 2. Financial disclosure & funder role | The following statement has been added to the cover letter and  will be updated in the online submission: *"The funders had no role*  *in study design, data collection and analysis, decision to publish,*  *or preparation of the manuscript."* |
| 3. Data availability | Data has been deposited in Zenodo and DOI generated. |
| 4. Figures | All figures have been removed from the manuscript file and  uploaded separately as high-resolution TIFF files. |

B. **Response to Reviewer Comments**

**Reviewer #1**

| Comment | Response | Location in revised manuscript |
| --- | --- | --- |
| 1.Revise title to include multi-site. | Title changed to: "Female sexual dysfunction in premenopausal women with diabetes mellitus in northern Nigeria: a cross-sectional study from two tertiary hospitals examining associations with glycaemic control and reproductive hormones" | Title page: Line 1,2,3 |
| 2. Define FSD and pathophysiological link in abstract and introduction | Added definition of FSD (disturbances in desire, arousal, orgasm, pain) and pathophysiological mechanisms (chronic hyperglycaemia, microvascular disease, neuropathy, hormonal disturbances) in both abstract and introduction. | Abstract (lines 28-30); Introduction (lines 76-78) |
| 3. State exact hormones measured | Added explicit list: "Follicle-stimulating hormone (FSH), luteinizing hormone (LH), oestrogen, testosterone, prolactin, and luteal-phase progesterone" | Abstract (lines 38-39); Methods (lines 183-184) |
| 4. In abstract conclusion, state which hormones not significantly affected and which FSFI domains affected | Revised conclusion: “Suboptimal glycaemic control was associated with FSD and with lower orgasm and satisfaction scores, while reproductive hormonal measures were not associated with FSD.” | Abstract (lines 50-53) |
| 5. Define IDF first usage. | Added definition: "International Diabetes Federation (IDF)" | Introduction: Line 62 |
| 6. Provide global, African, and Sub-Saharan African FSD prevalence data | Added prevalence data. | Introduction: Lines 93-99 |
| 7. Better description of blood sample collection | Added details: venous blood (5mL for hormones, 3mL for HbA1c), equipment (Cobas e411 analyser for hormones, Bio-Rad D-10 for HbA1c), storage (−20°C). | Methods: Lines 186-198 |
| 8. Expand discussion with comparisons to related works | Expanded discussion to compare findings, with explanations for differences (population characteristics, sample size, cultural factors) | Discussion: lines 383-387 |
| 9. Revise conclusion to state only observed findings | Conclusion revised to focus strictly on findings: prevalence (51.7%), association with suboptimal glycaemic control, specific domains (orgasm, satisfaction), and lack of association with reproductive hormones | Conclusion (lines 442-446) |

C. **Response to Reviewer #2**

| **Comment** | **Response** | **Location** |
| --- | --- | --- |
| 1.Cite IDF 2025 instead of 2021 | Updated reference to IDF Diabetes Atlas 2025 (11th edition) | Reference #2 |
| 2. Clarify "3.9 million adults" – is this Nigeria? | Clarified: "In Nigeria, an estimated 3.9 million adults had diabetes in 2019..." | Introduction: line 65 |
| **3.** Reference #5 (Uloko 2018) cannot support a 2024 statement | Corrected. Removed inappropriate citation and retained the 2024 Olamoyegun meta-analysis as the primary source for current prevalence | Introduction: lines 66-68 |
| 4. Justify convenience sampling | Added justification: convenience sampling was used to reduce investigator-driven selection, feasible within routine clinical care, and consecutive eligible women were invited to minimise bias | Methods: Lines 127-130 |
| 5. Justify age limit of 45 years | Age limit of 45 years was retained as it aligns with WHO definition of premenopausal women and ensures regular menstrual cycles, though Nigerian studies report menopause at 48-49 years, the 45-year cutoff provides a conservative buffer to exclude perimenopausal women with irregular cycles**.** | Methods: Line 135 |
| 6. Exclude patients with recent oophorectomy? | Added to exclusion criteria: Women who had undergone bilateral oophorectomy. | Method:  Line 139 |
| **7.** Reference for hormonal classification | Added reference: 24. Burtis CA, Ashwood ER, Bruns DE. Tietz Textbook of Clinical Chemistry and Molecular Diagnostics. 6th ed. Elsevier; 2018. | Method: Line 190 |
| 8. How were DM complications assessed? | Added: Diabetes-related complications (nephropathy, neuropathy, retinopathy) were assessed through review of medical records, participant self-report of diagnosed complications, clinical examination findings and investigations. | Method: Lines 199-205 |
| 9. Explain inconsistency: higher median HbA1c in FSD-negative vs positive (Table 2) | Added explanation in Results: "Although the median HbA1c was higher in the FSD-negative group (9.1% vs 8.3%), this difference was not statistically significant (p=0.5). The association between suboptimal glycaemic control and FSD emerged only after adjusting for confounders in multivariable analysis, suggesting that other factors (e.g., education, BMI, comorbidities) modified the crude relationship." | Results: Lines 259-263 |
| 10. Justify including both categorical HbA1c and continuous HbA1c in same model | Clarified: continuous HbA1c was included as a confounder (not collinear with categorical glycaemic control) to account for residual variation within the optimal/suboptimal categories. Alternative models using only categorical HbA1c yielded similar results. | Statistical Analysis: Lines 234-241) |
| 11. Check line 248-250 for clarity (repetition) | Repetition removed and modified | Discussion: Lines 333-334 |
| 12. Line 262-264: statement about "twice as often" requires non-DM control group | Addressed: The absence of a control group was stated as a limitation of the study. | Discussion/ Limitation: Lines 410-412 |
| 13. Expand discussion on glycaemic control and FSD | Added detailed mechanistic discussion: chronic hyperglycaemia → advanced glycation end-products → oxidative stress → endothelial dysfunction → impaired genital blood flow and clitoral/ vaginal innervation → orgasmic and satisfaction difficulties. | Discussion: Lines 360-364 |
| 14. Clarify DAG picture | Figure 3 has been re-created with higher resolution and clearer labels | Figure 3 |

D. **Response to Reviewer #3**

| **Comment** | **Response** | **Location** |
| --- | --- | --- |
| 1. Modify exclusion criteria formatting | Corrected to: "a) were pregnant at the time of recruitment; b) had reached menopause, defined as the absence of menstrual bleeding for 12 consecutive months." | Methods: Lines 137-138 |
| **2.** Clarify HbA1c measurement: previous results or new collection? | HbA1c was measured from venous blood samples collected on the day of recruitment for all participants. (Previous Hba1c results not used). | Methods: Lines 193-196 |
| **3.** Expand study procedure for reproducibility | Added detailed procedure: participants attended two visits (visit 1: consent, questionnaire, HbA1c, scheduling; visit 2: phase-specific blood draw). Follow-up calls were made to remind participants of appointment dates. | Methods: Lines 162-168 |
| **4.** Ethics: what happened to participants with positive FSD screen? | Added: "Participants who screened positive for FSD and those with hormonal imbalances were referred to the hospital's Family Medicine and Endocrinology clinics for further evaluation and management, including sexual counselling and endocrine optimisation, as part of standard care." | Ethics: Lines 145-149 |
| **5.** Explain "missing" values in Table 1 | Added footnote to Table 1: "Missing values occurred due to participants declining specific questions or had incomplete records. | Table 1 footnote |
| **6.** Table 1 too long – split into two tables | Table 1 split into: Table 1a (Sociodemographic characteristics) and Table 1b (Clinical and DM-related characteristics). | Tables 1A and 1B |
| **7.** Lines 248-250: repetition of results | Removed repetitive sentence "a similar proportion (51.7%) screened positive on the FSFI" that duplicated the first sentence | Discussion: Lines 333-334 |
| 8. Line 251: do not restate AOR in discussion without interpretation | Changed to: "Suboptimal glycaemic control was associated with nearly three-fold higher odds of screening positive for FSD after adjustment for confounders. | Discussion: 335-337 |
|  |  |  |

We believe these revisions have substantially strengthened the manuscript and addressed all concerns raised. We look forward to your positive decision.

Corresponding author: Dr. Fatima M. Damagum
Email: drfatima0984@gmail.com
