# Supplementary material for: Female sexual dysfunction in premenopausal women with diabetes mellitus in northern Nigeria: A cross-sectional study from two tertiary hospitals examining associations with glycaemic control and reproductive hormones
Source: PLoS One. 2026 Nov 2;21(11):e0361503. doi: 10.1371/journal.pone.0361503 (PMC13726980; doi:10.1371/journal.pone.0361503)
Supplement: Peer review history

# Peer review: Decision Letter 0

**DOI:** 10.1371/journal.pone.0361503.r001

**Authors:** Roland Eghoghosoa Akhigbe

14 May 2026

Dear Dr. Damagum,

Thank you for submitting your manuscript to PLOS ONE. After careful consideration, we feel that it has merit but does not fully meet PLOS ONE’s publication criteria as it currently stands. Therefore, we invite you to submit a revised version of the manuscript that addresses the points raised during the review process.

Please submit your revised manuscript by Jun 28 2026 11:59PM. If you will need more time than this to complete your revisions, please reply to this message or contact the journal office at plosone@plos.org. When you're ready to submit your revision, log on to https://www.editorialmanager.com/pone/ and select the 'Submissions Needing Revision' folder to locate your manuscript file.

- A letter that responds to each point raised by the academic editor and reviewer(s). You should upload this letter as a separate file labeled 'Response to Reviewers'.
- A marked-up copy of your manuscript that highlights changes made to the original version. You should upload this as a separate file labeled 'Revised Manuscript with Track Changes'.
- An unmarked version of your revised paper without tracked changes. You should upload this as a separate file labeled 'Manuscript'.

If you would like to make changes to your financial disclosure, please include your updated statement in your cover letter. Guidelines for resubmitting your figure files are available below the reviewer comments at the end of this letter.

If applicable, we recommend that you deposit your laboratory protocols in protocols.io to enhance the reproducibility of your results. Protocols.io assigns your protocol its own identifier (DOI) so that it can be cited independently in the future. For instructions see: https://journals.plos.org/plosone/s/submission-guidelines#loc-laboratory-protocols. Additionally, PLOS ONE offers an option for publishing peer-reviewed Lab Protocol articles, which describe protocols hosted on protocols.io. Read more information on sharing protocols at https://plos.org/protocols?utm_medium=editorial-email&utm_source=authorletters&utm_campaign=protocols.

As the corresponding author, your ORCID iD is verified in the submission system and will appear in the published article. PLOS supports the use of ORCID, and we encourage all coauthors to register for an ORCID iD and use it as well. Please encourage your coauthors to verify their ORCID iD within the submission system before final acceptance, as unverified ORCID iDs will not appear in the published article. *Only*  the individual author can complete the verification step; PLOS staff *cannot*  verify ORCID iDs on behalf of authors.

We look forward to receiving your revised manuscript.

Kind regards,

Roland Eghoghosoa Akhigbe

Academic Editor

PLOS One

**Journal Requirements:**

When submitting your revision, we need you to address these additional requirements.

1. Please ensure that your manuscript meets PLOS ONE's style requirements, including those for file naming. The PLOS ONE style templates can be found at

https://journals.plos.org/plosone/s/file?id=wjVg/PLOSOne_formatting_sample_main_body.pdf and

https://journals.plos.org/plosone/s/file?id=ba62/PLOSOne_formatting_sample_title_authors_affiliations.pdf

2. Thank you for stating the following financial disclosure:

“This research was supported by the Fogarty International Center and the Office of Research on Women's Health (ORWH), of the National Institutes for Health under Award Number D43 TW010543. FD recieved this award. The content is solely the responsibility of the authors and does not necessarily represent the official views of the National Institutes of Health.”

Please state what role the funders took in the study.  If the funders had no role, please state: "The funders had no role in study design, data collection and analysis, decision to publish, or preparation of the manuscript."

If this statement is not correct you must amend it as needed.

Please include this amended Role of Funder statement in your cover letter; we will change the online submission form on your behalf.

3. In the online submission form, you indicated that “Data is available upon request”.

All PLOS journals now require all data underlying the findings described in their manuscript to be freely available to other researchers, either 1. In a public repository, 2. Within the manuscript itself, or 3. Uploaded as supplementary information.

This policy applies to all data except where public deposition would breach compliance with the protocol approved by your research ethics board. If your data cannot be made publicly available for ethical or legal reasons (e.g., public availability would compromise patient privacy), please explain your reasons on resubmission and your exemption request will be escalated for approval.

4. Please remove your figures from within your manuscript file, leaving only the individual TIFF/EPS image files, uploaded separately. These will be automatically included in the reviewers’ PDF.

5. If the reviewer comments include a recommendation to cite specific previously published works, please review and evaluate these publications to determine whether they are relevant and should be cited. There is no requirement to cite these works unless the editor has indicated otherwise.

[Note: HTML markup is below. Please do not edit.]

Reviewers' comments:

Reviewer's Responses to Questions

**Comments to the Author**

1. Is the manuscript technically sound, and do the data support the conclusions?

Reviewer #1: Yes

Reviewer #2: Partly

Reviewer #3: Yes

**********

2. Has the statistical analysis been performed appropriately and rigorously?

Reviewer #1: Yes

Reviewer #2: Yes

Reviewer #3: Yes

**********

3. Have the authors made all data underlying the findings in their manuscript fully available?

The PLOS Data policy

Reviewer #1: No

Reviewer #2: No

Reviewer #3: Yes

**********

4. Is the manuscript presented in an intelligible fashion and written in standard English?

Reviewer #1: Yes

Reviewer #2: Yes

Reviewer #3: Yes

**********

Reviewer #1: since two centers were involved, authors should revise the title to include multi sites

Authors should define FSD and state the pathophysiological link between FSD, and DM, hormonal, clinical implications and how its important both in abstract and introduction section. Authors should state the exact hormones that were measured. In abstract conclusion, authors should state the exact hormones that were not significantly affected by FSD as well as the exact domains of sexual function affected

Authors should define IDF first time usage line 62. Authors should provide some data on prevalence of FSD globally, Africa and sub-Sahara africa

A case-control study comparing FSD among DM and normal female population would have given a better inference. Authors should give better description of how blood samples were collected and analyzed, equipment used, types and brand, volume of blood and timepoint for the blood collection.

Tables and figures should be presented in a standard format

Authors should expatiate more on the discussion by stating observed findings, compare with related works and state similarities or differences and reasons for the observed differences/similarities.

Conclusion should be revised to state only observed findings

Reviewer #2: This is a well-conceived, clinically relevant study addressing an under-researched area—female sexual dysfunction (FSD) in women with diabetes in sub-Saharan Africa

INTRODUCTION

Line 55: It will be more appropriate to cite IDF 2025 as it has been available for more than 1 year

Line 59-60: The 3.9 million adults needed clarification. Is it for Nigeria?

Line 60-61: A systematic review looks at a combination of other researchers, so it is not necessarily reflective of current prevalence. Also your reference number 5 was published is 2018, therefore not possible to have been used as a reference here. Kindly change as it is not reflective. Your reference 6 refers to systematic review in female sexual dysfunction, how is related to the points made here?

METHODS

How did you determine that the prevalence of

How did you ensure some form of probability sampling? Why was convenience sampling used?

Why the age limit of 45 years especially as a number of Nigerian studies report menopause between 48-49 years

Did you exclude patients who had recent oophorectomy?

Line 151-152: Do you have a reference for this?

How were DM complications assessed ?

RESULTS

Although suboptimal glycaemic control was associated with FSD in the adjusted model, the crude HbA1c distribution showed a higher median HbA1c among FSD-negative women. The authors should explain this apparent inconsistency and present the proportion of women with HbA1c >7% by FSD status. They should also justify including both categorical glycaemic control and continuous HbA1c in the same regression model, or consider alternative models using HbA1c either continuously or categorically, but not both simultaneously.

DISCUSSION

Check Line 248 -249 for clarity

Line 262-264 does not apply to your study as there were no controls. Did you assess other non-DM patients in your study to arrive at this conclusion?

Your discussion failed to explore the relationship between glycemic control and FSD into detail. Kindl expand on that. Also include findings from other studies around the subject.

OTHERS

Your DAG picture is not clear

Reviewer #3: Comments

1.Eligibility criteria: line 116 and 117: the exclusion criteria need to be modified to reflect the omitted count. “a) were pregnant at the time of recruitment. b) had reached menopause, defined as the absence of menstrual bleeding for 12 consecutive months.”

2.Data collection: What do you mean when you say, in lines 147-149: “Glycaemic control was assessed using glycated Haemoglobin (HbA1c) levels obtained from venous blood samples collected during clinic visits.” It is important to state whether blood samples were collected at this time from all participants for A1c, whether previous A1c test results (especially those done within 3 months of the recruitment date) were used, or whether a combination was used.

3.For reproducibility of the work, the study procedure may need to be expanded. For instance, it is important to know whether there was a follow-up of participants who needed to return with the results of their tests (hormonal profiles or A1c).

4.Ethics: It is also important to know what happened to participants with positive FSFI screen and hormonal imbalance in your study. This should be mentioned in the ethics statement.

5.What accounts for the “missing” seen in Table 1? This explanation should come as a footnote. Again, Table 1 appears to be too long; creating two tables, one for sociodemographics and another for clinical characteristics, would be helpful, if it does not exceed the recommended limit for the journal.

6.Discussion, Lines 248-250: There appears to be a repetition of the results here. “More than half (51.7%) of the participants screened positive for FSD on the FSFI; a similar proportion (51.7%) screened positive on the FSFI.” Please clarify.

7.Line 251: There is no need to restate the results, e.g., (adjusted odds ratio [AOR] 2.80, 95% CI 1.05–7.72) in the discussion section. The reader needs to know what it means, how it differs from current evidence and how it impacts practice, policy and future research.

**********

what does this mean?). If published, this will include your full peer review and any attached files.

If you choose “no”, your identity will remain anonymous but your review may still be made public.

**Do you want your identity to be public for this peer review?**  For information about this choice, including consent withdrawal, please see our Privacy Policy

Reviewer #1: **Yes:**  Samuel Asamoah Sakyi

Reviewer #2: No

Reviewer #3: **Yes:**  Godpower Chinedu Michael

**********

[NOTE: If reviewer comments were submitted as an attachment file, they will be attached to this email and accessible via the submission site. Please log into your account, locate the manuscript record, and check for the action link "View Attachments". If this link does not appear, there are no attachment files.]

To ensure your figures meet our technical requirements, please review our figure guidelines: https://journals.plos.org/plosone/s/figures

You may also use PLOS’s free figure tool, NAAS, to help you prepare publication quality figures: https://journals.plos.org/plosone/s/figures#loc-tools-for-figure-preparation.

NAAS will assess whether your figures meet our technical requirements by comparing each figure against our figure specifications.

# Author comment: Author response to Decision Letter 1

**DOI:** 10.1371/journal.pone.0361503.r002

24 May 2026

Dear Academic Editor,

Thank you for the opportunity to revise our manuscript. We are grateful for the thoughtful and constructive feedback provided by the reviewers. We have addressed all comments point-by-point in the table below and revised the manuscript accordingly. All changes are highlighted in the revised manuscript using track changes.

A. Response to Journal Requirements

Requirement Action Taken

1.PLOS ONE style requirements The manuscript has been formatted according to the PLOS ONE templates, including file naming, headings, and reference style.

2. Financial disclosure & funder role The following statement has been added to the cover letter and

will be updated in the online submission: "The funders had no role

in study design, data collection and analysis, decision to publish,

or preparation of the manuscript."

3. Data availability Data has been deposited in Zenodo and DOI generated.

4. Figures All figures have been removed from the manuscript file and

uploaded separately as high-resolution TIFF files.

B. Response to Reviewer Comments

Reviewer #1

Comment Response Location in revised manuscript

1.Revise title to include multi-site. Title changed to: "Female sexual dysfunction in premenopausal women with diabetes mellitus in northern Nigeria: a cross-sectional study from two tertiary hospitals examining associations with glycaemic control and reproductive hormones" Title page: Line 1,2,3

2. Define FSD and pathophysiological link in abstract and introduction Added definition of FSD (disturbances in desire, arousal, orgasm, pain) and pathophysiological mechanisms (chronic hyperglycaemia, microvascular disease, neuropathy, hormonal disturbances) in both abstract and introduction. Abstract (lines 28-30); Introduction (lines 76-78)

3. State exact hormones measured Added explicit list: "Follicle-stimulating hormone (FSH), luteinizing hormone (LH), oestrogen, testosterone, prolactin, and luteal-phase progesterone" Abstract (lines 38-39); Methods (lines 183-184)

4. In abstract conclusion, state which hormones not significantly affected and which FSFI domains affected Revised conclusion: “Suboptimal glycaemic control was associated with FSD and with lower orgasm and satisfaction scores, while reproductive hormonal measures were not associated with FSD.” Abstract (lines 50-53)

5. Define IDF first usage. Added definition: "International Diabetes Federation (IDF)" Introduction: Line 62

6. Provide global, African, and Sub-Saharan African FSD prevalence data Added prevalence data. Introduction: Lines 93-99

7. Better description of blood sample collection Added details: venous blood (5mL for hormones, 3mL for HbA1c), equipment (Cobas e411 analyser for hormones, Bio-Rad D-10 for HbA1c), storage (−20°C). Methods: Lines 186-198

8. Expand discussion with comparisons to related works Expanded discussion to compare findings, with explanations for differences (population characteristics, sample size, cultural factors) Discussion: lines 383-387

9. Revise conclusion to state only observed findings Conclusion revised to focus strictly on findings: prevalence (51.7%), association with suboptimal glycaemic control, specific domains (orgasm, satisfaction), and lack of association with reproductive hormones Conclusion (lines 442-446)

C. Response to Reviewer #2

Comment Response Location

1.Cite IDF 2025 instead of 2021 Updated reference to IDF Diabetes Atlas 2025 (11th edition) Reference #2

2. Clarify "3.9 million adults" – is this Nigeria? Clarified: "In Nigeria, an estimated 3.9 million adults had diabetes in 2019..." Introduction: line 65

3. Reference #5 (Uloko 2018) cannot support a 2024 statement Corrected. Removed inappropriate citation and retained the 2024 Olamoyegun meta-analysis as the primary source for current prevalence Introduction: lines 66-68

4. Justify convenience sampling Added justification: convenience sampling was used to reduce investigator-driven selection, feasible within routine clinical care, and consecutive eligible women were invited to minimise bias Methods: Lines 127-130

5. Justify age limit of 45 years Age limit of 45 years was retained as it aligns with WHO definition of premenopausal women and ensures regular menstrual cycles, though Nigerian studies report menopause at 48-49 years, the 45-year cutoff provides a conservative buffer to exclude perimenopausal women with irregular cycles.

Methods: Line 135

6. Exclude patients with recent oophorectomy? Added to exclusion criteria: Women who had undergone bilateral oophorectomy. Method:

Line 139

7. Reference for hormonal classification Added reference: 24. Burtis CA, Ashwood ER, Bruns DE. Tietz Textbook of Clinical Chemistry and Molecular Diagnostics. 6th ed. Elsevier; 2018. Method: Line 190

8. How were DM complications assessed? Added: Diabetes-related complications (nephropathy, neuropathy, retinopathy) were assessed through review of medical records, participant self-report of diagnosed complications, clinical examination findings and investigations. Method: Lines 199-205

9. Explain inconsistency: higher median HbA1c in FSD-negative vs positive (Table 2) Added explanation in Results: "Although the median HbA1c was higher in the FSD-negative group (9.1% vs 8.3%), this difference was not statistically significant (p=0.5). The association between suboptimal glycaemic control and FSD emerged only after adjusting for confounders in multivariable analysis, suggesting that other factors (e.g., education, BMI, comorbidities) modified the crude relationship." Results: Lines 259-263

10. Justify including both categorical HbA1c and continuous HbA1c in same model Clarified: continuous HbA1c was included as a confounder (not collinear with categorical glycaemic control) to account for residual variation within the optimal/suboptimal categories. Alternative models using only categorical HbA1c yielded similar results. Statistical Analysis: Lines 234-241)

11. Check line 248-250 for clarity (repetition) Repetition removed and modified Discussion: Lines 333-334

12. Line 262-264: statement about "twice as often" requires non-DM control group Addressed: The absence of a control group was stated as a limitation of the study. Discussion/ Limitation: Lines 410-412

13. Expand discussion on glycaemic control and FSD Added detailed mechanistic discussion: chronic hyperglycaemia → advanced glycation end-products → oxidative stress → endothelial dysfunction → impaired genital blood flow and clitoral/ vaginal innervation → orgasmic and satisfaction difficulties. Discussion: Lines 360-364

14. Clarify DAG picture Figure 3 has been re-created with higher resolution and clearer labels Figure 3

D. Response to Reviewer #3

Comment Response Location

1. Modify exclusion criteria formatting Corrected to: "a) were pregnant at the time of recruitment; b) had reached menopause, defined as the absence of menstrual bleeding for 12 consecutive months." Methods: Lines 137-138

2. Clarify HbA1c measurement: previous results or new collection? HbA1c was measured from venous blood samples collected on the day of recruitment for all participants. (Previous Hba1c results not used). Methods: Lines 193-196

3. Expand study procedure for reproducibility Added detailed procedure: participants attended two visits (visit 1: consent, questionnaire, HbA1c, scheduling; visit 2: phase-specific blood draw). Follow-up calls were made to remind participants of appointment dates. Methods: Lines 162-168

4. Ethics: what happened to participants with positive FSD screen? Added: "Participants who screened positive for FSD and those with hormonal imbalances were referred to the hospital's Family Medicine and Endocrinology clinics for further evaluation and management, including sexual counselling and endocrine optimisation, as part of standard care." Ethics: Lines 145-149

5. Explain "missing" values in Table 1 Added footnote to Table 1: "Missing values occurred due to participants declining specific questions or had incomplete records. Table 1 footnote

6. Table 1 too long – split into two tables Table 1 split into: Table 1a (Sociodemographic characteristics) and Table 1b (Clinical and DM-related characteristics). Tables 1A and 1B

7. Lines 248-250: repetition of results Removed repetitive sentence "a similar proportion (51.7%) screened positive on the FSFI" that duplicated the first sentence Discussion: Lines 333-334

8. Line 251: do not restate AOR in discussion without interpretation Changed to: "Suboptimal glycaemic control was associated with nearly three-fold higher odds of screening positive for FSD after adjustment for confounders. Discussion: 335-337

We believe these revisions have substantially strengthened the manuscript and addressed all concerns raised. We look forward to your positive decision.

Corresponding author: Dr. Fatima M. Damagum

Email: drfatima0984@gmail.com

Attachment Submitted filename: Response to Reviewers.docx

# Peer review: Decision Letter 1

**DOI:** 10.1371/journal.pone.0361503.r003

**Authors:** George Vousden

12 Oct 2026

Female sexual dysfunction in premenopausal women with diabetes mellitus in northern Nigeria: a cross-sectional study from two tertiary hospitals examining associations with glycaemic control and reproductive hormones

PONE-D-26-16614R1

Dear Dr. Damagum,

We’re pleased to inform you that your manuscript has been judged scientifically suitable for publication and will be formally accepted for publication once it meets all outstanding technical requirements.

Within one week, you’ll receive an e-mail detailing the required amendments. When these have been addressed, you’ll receive a formal acceptance letter and your manuscript will be scheduled for publication.

An invoice will be generated when your article is formally accepted. Please note, if your institution has a publishing partnership with PLOS and your article meets the relevant criteria, all or part of your publication costs will be covered. Please make sure your user information is up-to-date by logging into Editorial Manager at Editorial Manager® and clicking the ‘Update My Information' link at the top of the page. For questions related to billing, please contact billing support.

If your institution or institutions have a press office, please notify them about your upcoming paper to help maximize its impact. If they’ll be preparing press materials, please inform our press team as soon as possible -- no later than 48 hours after receiving the formal acceptance. Your manuscript will remain under strict press embargo until 2 pm Eastern Time on the date of publication. For more information, please contact onepress@plos.org.

Kind regards,

George Vousden

Staff Editor

PLOS One

Additional Editor Comments (optional):

Reviewers' comments:

Reviewer's Responses to Questions

**Comments to the Author**

Reviewer #2: All comments have been addressed

Reviewer #3: All comments have been addressed

**********

2. Is the manuscript technically sound, and do the data support the conclusions?

Reviewer #2: Yes

Reviewer #3: Yes

**********

3. Has the statistical analysis been performed appropriately and rigorously?

Reviewer #2: Yes

Reviewer #3: Yes

**********

4. Have the authors made all data underlying the findings in their manuscript fully available?

The PLOS Data policy

Reviewer #2: Yes

Reviewer #3: Yes

**********

5. Is the manuscript presented in an intelligible fashion and written in standard English?

Reviewer #2: Yes

Reviewer #3: Yes

**********

Reviewer #2: The authors have addressed all comments satisfactorily. This has therefore improved on the quality of this manuscript.

Reviewer #3: My queries on the Eligibility criteria, data collection, the need to expand the study procedure, ethics, duplicated sentences in the discussion section have all been addressed.

**********

what does this mean?). If published, this will include your full peer review and any attached files.

If you choose “no”, your identity will remain anonymous but your review may still be made public.

**Do you want your identity to be public for this peer review?**  For information about this choice, including consent withdrawal, please see our Privacy Policy

Reviewer #2: No

Reviewer #3: **Yes:**  Godpower Chinedu Michael

**********

# Editor report: Acceptance letter

**DOI:** 10.1371/journal.pone.0361503.r004

**Authors:** George Vousden

PONE-D-26-16614R1

PLOS One

Dear Dr. Damagum,

I'm pleased to inform you that your manuscript has been deemed suitable for publication in PLOS One. Congratulations! Your manuscript is now being handed over to our production team.

At this stage, our production department will prepare your paper for publication. This includes ensuring the following:

* All references, tables, and figures are properly cited

* All relevant supporting information is included in the manuscript submission,

* There are no issues that prevent the paper from being properly typeset

You will receive further instructions from the production team, including instructions on how to review your proof when it is ready. Please keep in mind that we are working through a large volume of accepted articles, so please give us a few days to review your paper and let you know the next and final steps.

Lastly, if your institution or institutions have a press office, please let them know about your upcoming paper now to help maximize its impact. If they'll be preparing press materials, please inform our press team within the next 48 hours. Your manuscript will remain under strict press embargo until 2 pm Eastern Time on the date of publication. For more information, please contact onepress@plos.org.

You will receive an invoice from PLOS for your publication fee after your manuscript has reached the completed accept phase. If you receive an email requesting payment before acceptance or for any other service, this may be a phishing scheme. Learn how to identify phishing emails and protect your accounts at https://explore.plos.org/phishing.

If we can help with anything else, please email us at customercare@plos.org.

Thank you for submitting your work to PLOS One and supporting open access.

Kind regards,

PLOS One Editorial Office Staff

on behalf of

Dr. George Vousden

Staff Editor

PLOS One
